# Short-Term Outcomes of Refractory Diabetic Macular Edema Switch From Ranibizumab to Dexamethasone Implant and the Influential Factors: A Retrospective Real World Experience

**DOI:** 10.3389/fmed.2021.649979

**Published:** 2021-04-30

**Authors:** Ning-Yi Hsia, Chun-Ju Lin, Huan-Sheng Chen, Cheng-Hsien Chang, Henry Bair, Chun-Ting Lai, Jane-Ming Lin, Wen-Lu Chen, Peng-Tai Tien, Wen-Chuan Wu, Yi-Yu Tsai

**Affiliations:** ^1^Department of Ophthalmology, Eye Center, China Medical University Hospital, Taichung, Taiwan; ^2^School of Medicine, College of Medicine, China Medical University, Taichung, Taiwan; ^3^Department of Optometry, Asia University, Taichung, Taiwan; ^4^An-Shin Dialysis Center, NephroCare Ltd., Fresenius Medical Care, Taichung, Taiwan; ^5^School of Medicine, Stanford University, Stanford, CA, United States; ^6^Graduate Institute of Clinical Medical Science, China Medical University, Taichung, Taiwan

**Keywords:** diabetic macular edema, intravitreal dexamethasone implant, intravitreal ranibizumab, ozurdex, refractory diabetic macular edema

## Abstract

**Introduction:** To evaluate the effectiveness and safety of intravitreal dexamethasone (DEX) implants in refractory diabetic macular edema (DME) treated by intravitreal ranibizumab.

**Materials and Methods:** We retrospectively analyzed DME patients who received DEX implant treatment after being refractory to at least 3 monthly intravitreal ranibizumab injections. The main outcomes were best-corrected visual acuity (BCVA), central retinal thickness (CRT), and intraocular pressure (IOP).

**Results:** Twenty-nine eyes of 26 patients who had previously received an average of 8.1 ± 4.4 ranibizumab injections were included. Patients received between one and three DEX implants during 12.4 ± 7.4 months of follow-up. The mean final CRT significantly decreased from 384.4 ± 114.4 μm at baseline to 323.9 ± 77.7 μm (*p* = 0.0249). The mean final BCVA was 51.4 ± 21.3 letters, which was not significant compared to baseline (44.9 ± 30.2 letters, *p* = 0.1149). Mean IOP did not increase significantly. All patients tolerated the treatment well without serious adverse events. Higher baseline CRT and worse BCVA correlated with better therapeutic responses.

**Conclusion:** Switching to DEX implant is feasible and safe for treating patients of DME refractory to intravitreal ranibizumab in real world. Further larger-scale or multicenter studies would be conducted to explore different DEX treatment strategies for DME, such as first-line or early switch therapy, for better BCVA improvement.

## Introduction

Diabetes mellitus is one of the most important global health issues of the twenty-first century. At present, there are 425 million patients with diabetes worldwide, and this number is projected to reach 629 million by 2045 (1). Diabetic retinopathy, a microvascular complication of diabetes, has an estimated prevalence of 34.6% among patients with diabetes. Diabetic macular edema (DME), a manifestation of diabetic retinopathy, develops in ~6.8% of patients with diabetes and is a major cause of visual loss in this population (2).

Hyperglycemia in diabetes increases oxidative stress, inflammation, and vascular dysfunction. Oxidative stress and inflammation induce the upregulation of growth factors, such as vascular endothelial growth factor (VEGF) and cytokines, which contribute to the breakdown of the blood-retinal barrier (BRB) by disrupting the integrity of retinal vascular endothelial cell tight junctions and increasing vascular permeability (3). The ensuing fluid accumulation, in addition to the persistent presence of inflammatory factors, causes dysfunction of the inner nuclear layer and subsequent development of DME (4).

VEGF antagonists are frequently used as intravitreal treatments for DME, as several studies reveal that patients with DME had favorable visual and anatomic responses to ranibizumab (5, 6). However, there are still patients who, after a favorable initial response to anti-VEGF agents, show decreased responses over time and became resistant to further intravitreal injections. This may be a result of inflammatory mediators other than VEGF contributing to the persistence of DME (7). Increasing dosages of intravitreal injections are needed to control the disease. However, this carries an increased risk of complications and poor compliance (8).

Corticosteroids have been demonstrated to inhibit the expression of VEGF and other inflammatory factors, thus reinforcing the BRB. The biodegradable intravitreal dexamethasone (DEX) implant provides sustained release of the anti-inflammatory corticosteroid dexamethasone into the vitreous. DEX implants have been identified as an effective treatment of DME and have recently been approved by the US Food and Drug Administration (FDA) (9–11). We thus conducted this study to investigate anatomic and functional improvements of DEX implant treatment in a group of patients with DME refractory to previous ranibizumab injections.

## Materials and Methods

This retrospective, non-comparative, consecutive case series study was approved by the Institutional Ethics Committee and conducted in compliance with the tenets of the Declaration of Helsinki. We retrospectively analyzed the eyes of patients with DME refractory to intravitreal ranibizumab treatment, were treated with DEX implant between August 2013 and October 2017. Informed oral and written consent was obtained from all patients. Before March 2020, Taiwan National Health Insurance scheme only reimbursed 3 initial plus 5 additional injections of ranibizumab for eligible patients with DME. No switch to DEX was allowed. Therefore, patients had to continue 3 to 8 ranibizumab injections unless they decide to pay for DEX out of pocket.

The inclusion criteria were as follows: (1) a diagnosis of DME (the presentation of choroidal neovascularization with macular edema, confirmed by fluorescein angiography and optical coherence tomography [OCT]); (2) a history of treatment with at least 3 monthly intravitreal ranibizumab injections, followed by increasing or persistent sub-retinal fluid or retinal edema on OCT; and (3) a CRT >250 μm. The criteria for treatment with DEX implant were the same as the retreatment criteria for ranibizumab regarding the presence of intraretinal or subretinal fluid.

We recorded general patient data including age, sex, laterality, medical history, glycated hemoglobin (HbA1c), best-corrected visual acuity (BCVA), central retinal thickness (CRT), intraocular pressure (IOP), and results of external ocular and slit-lamp examinations. Each patient underwent a thorough bilateral fundus examination by indirect ophthalmoscopy, fundus photography, fluorescein angiography, and spectral-domain OCT (Cirrus HD-OCT; Carl Zeiss Meditec, Inc., Dublin, CA) scans. Over the course of the treatment, patients received between one and three injections of DEX implant 0.7 mg (Ozurdex, Allergan, Inc, Irvine, CA). Before each DEX implantation, the topical antibiotic levofloxacin (Cravit, Santen Pharmaceutical Co., Osaka, Japan) was applied. Topical and subconjunctival anesthesia was achieved by 0.5% proparacaine hydrochloride (Alcaine, Alcon Pharmaceuticals, Puurs, Belgium) before surgery. Each eye was prepared in a sterile manner using 5% povidone/iodine. The DEX implant was inserted intravitreally via a pars plana puncture (3.5 mm away from the limbus). Application of levofloxacin eyedrops was prescribed four times a day for 1 week after the operation. Initial management with ranibizumab and the number of subsequent treatments with DEX implant were collected. All of the patients were scheduled for monthly follow-ups.

The main outcome measures included the mean change in CRT from baseline as measured by spectral-domain OCT and mean change in BCVA (approximate Early Treatment Diabetic Retinopathy Study [ETDRS] letter scores) from baseline during monthly follow-ups. Therefore, the outcome of DEX implant after ranibizumab was evaluated by analyzing changes in retinal anatomy and vision, with reference to patient characteristics and fundus findings. Safety was evaluated by recording complications and other adverse events during the follow-up period.

For statistical analyses, SAS 9.4 was used in this study. For comparison of cross-section data, one-way ANOVA was used for continuous data and Fisher's exact test was used for categorical data. For comparison of serial data, the principle of a generalized linear mixed model (GLMM) was applied using the GLIMMIX procedure in SAS. Generalized linear mixed model is actually a method with the same concept as repeated measured ANOVA for serial data comparison but is more flexible and tolerant of data completeness. Shapiro-Wilk test and Kolmogorov-Smirnov tests were both used for normality test with SAS procedure univariate.

## Results

### Study Population and Treatments

A total of 29 eyes of 26 patients with DME were included in this study. The study group comprised of 14 men and 12 women, and the mean age was 62.0 ± 9.1 (range 46–84) years. The mean baseline HbA1c was 7.5 ± 1.3 %. Before any treatment (baseline), the mean CRT was 384.4 ± 114.4 (range 248–727) μm (Table 1). After ranibizumab treatment, patients were followed up for an average of 12.4 ± 7.4 months. Prior to receiving DEX implant treatment, all patients had been treated with an average of 8.1 ± 4.4 (range 3–18) injections of intravitreal ranibizumab. The time between the last ranibizumab injection and the first DEX injection was a month. Each eye received an average of 1.3 ± 0.6 DEX implant (range 1–3) injections. Of the 29 study eyes, 23 eyes received only one DEX implant, four eyes received two DEX implants and two eyes received three DEX implants. The mean interval between DEX implant injections in the six eyes that received more than one injection was 5.93 ± 2.68 months (range 3.1 ~ 10.2 months). The follow-up period before DEX was 12.39 ± 7.44 months, the follow-up period after DEX was 7.43 ± 4.60 months, and the entire follow-up period was 19.82 ± 8.96 months.

**Table 1:** Baseline characteristics of the patients.

**Baseline characteristics**	**Mean ± SD or (%)**
Age (years)	62.0 ± 9.1
Gender (*n* = 26)	
Female	12 (46.2%)
Male	14 (53.8%)
Eyes (*n* = 29)	
OD	16 (55.2%)
OS	13 (44.8%)
Baseline BCVA (letter score)	44.9 ± 30.2
Baseline CRT (μm)	384.4 ± 114.4
Baseline IOP (mmHg)	14.9 ± 3.1
Lens status	
Phakic	20 (69%)
Pseudophakic	9 (31%)
Follow-up (months)	
Total	19.8 ± 9.0
Before ozurdex (anti-VEGF use)	12.4 ± 7.4
After ozurdex	7.4 ± 4.6

*BCVA, best-corrected visual acuity; CRT, central retina thickness; IOP, intraocular pressure; VEGF, vascular endothelial growth factor*.

### Anatomic Changes

**Figure 1:**
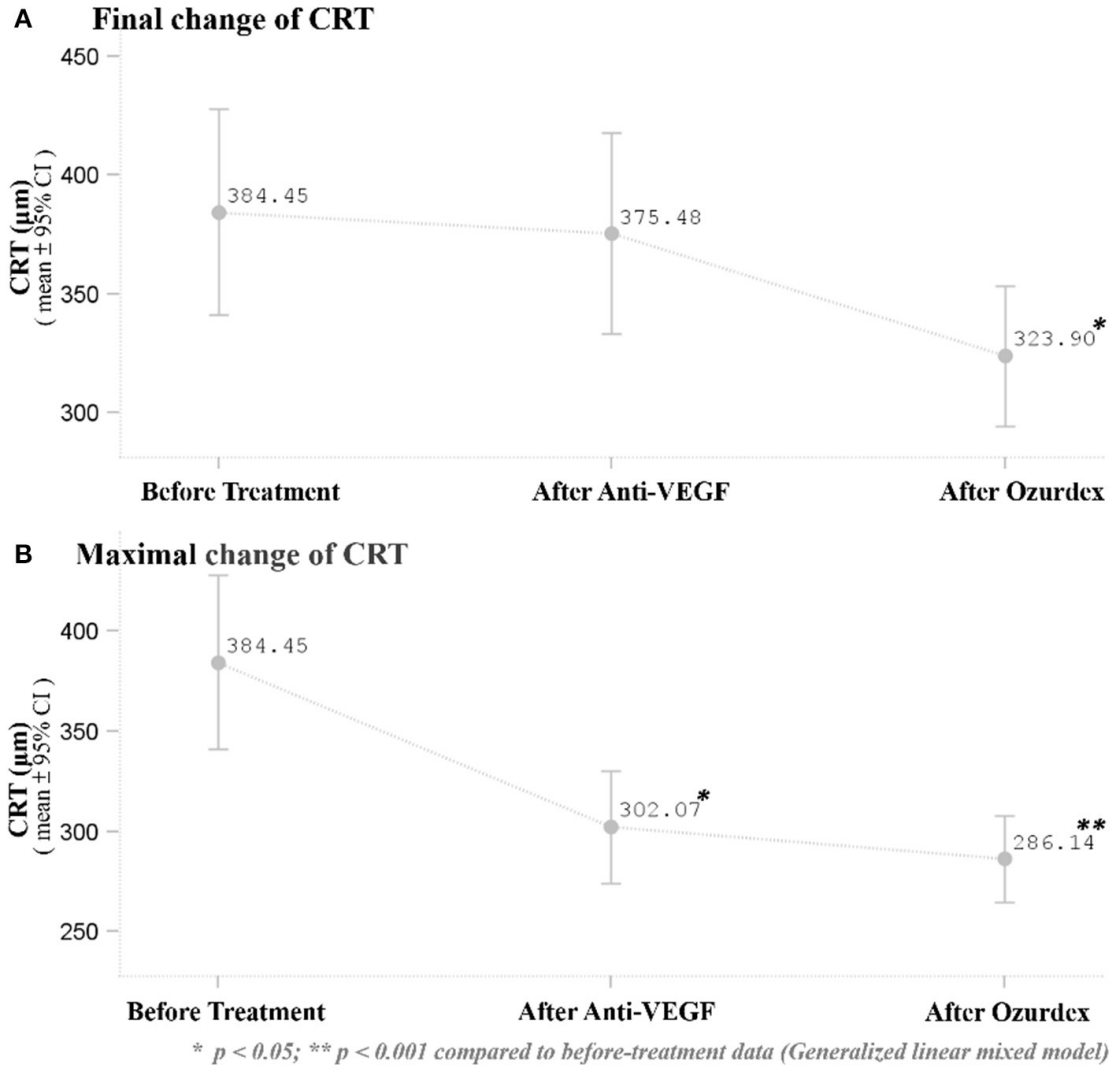
**(A)** Mean baseline and final CRT after the respective treatments. The mean final CRT after DEX implant treatment was significantly lower than the CRT (*p* = 0.0249) at baseline and 1 month after the last ranibizumab injection (*p* = 0.0265). **(B)** Mean best CRT (the lowest CRT value recorded during follow-up) at baseline and after the respective treatments. The mean best CRT after DEX implant treatment was significantly lower than the CRT (*p* < 0.0001) before treatment. *p < 0.05; **p < 0.001 compared to before-treatment data (Generalized linear mixed model).

Distribution of CRT thickness before DEX implant treatment were as follows: 16 eyes between 350 and 250 μm, 8 eyes between 450 and 350 μm, 3 eyes between 550 and 450 μm, 1 eye between 650 and 550 μm, and 1 eye >650 μm. All eyes showed anatomic improvement after switching to intravitreal DEX implant treatment, with significant postoperative changes in CRT as measured by OCT. After DEX implant treatment, the mean final CRT (323.9 ± 77.7, range 201–488 μm) was significantly lower than the baseline value (384.5 ± 114.4, range 248–727 μm) (*p* = 0.0249), and also significantly lower than the mean CRT at 1 month after the last injection of ranibizumab (375.5 ± 111.0, range 261–757 μm) (*p* = 0.0265; Figure 1A). The mean best CRT (the lowest CRT value recorded during follow-up)after ranibizumab treatment (302.1 ± 73.5, range 195–568 μm) was significantly lower than the baseline value (*p* = 0.0004), and it was maintained if not further improved after DEX implants (286.1 ± 56.3, range 172–414 μm) (*p* < 0.0001 compared to baseline; Figure 1B).

### Changes in Best-Corrected Visual Acuity

**Figure 2:**
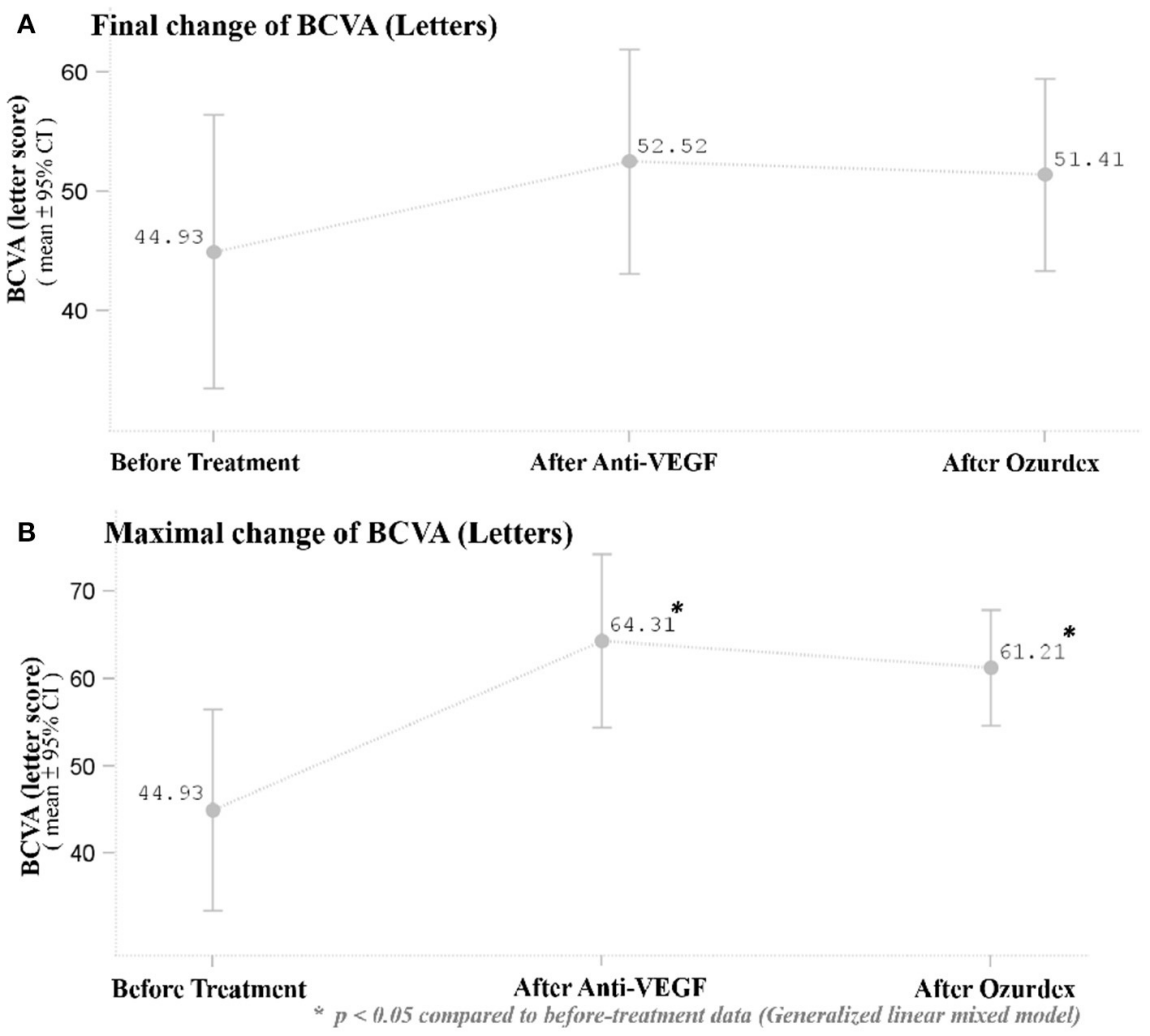
**(A)** Mean BCVA at baseline and after the respective treatments. There was no significant improvement in BCVA 1 month after the last ranibizumab and after DEX implant treatment. **(B)** Mean maximal BCVA (the highest letter score recorded during follow-up) at baseline and after respective treatments. The mean maximal BCVA after DEX implant treatment was significantly higher than baseline BCVA (*p* = 0.0022). *p < 0.05 compared to before-treatment data (Generalized linear mixed model).

After DEX implant treatment, the mean final BCVA (51.4 ± 21.3 letters) did not significantly improve as compared to baseline (44.9 ± 30.2 letters) (*p* = 0.1149; Figure 2A). However, the mean maximal BCVA (the highest letter score recorded during follow-up) after DEX implant (61.2 ± 17.4 letters) was significantly higher than baseline (*p* = 0.0022; Figure 2B).

### Predictors of Therapeutic Response

**Figure 3:**
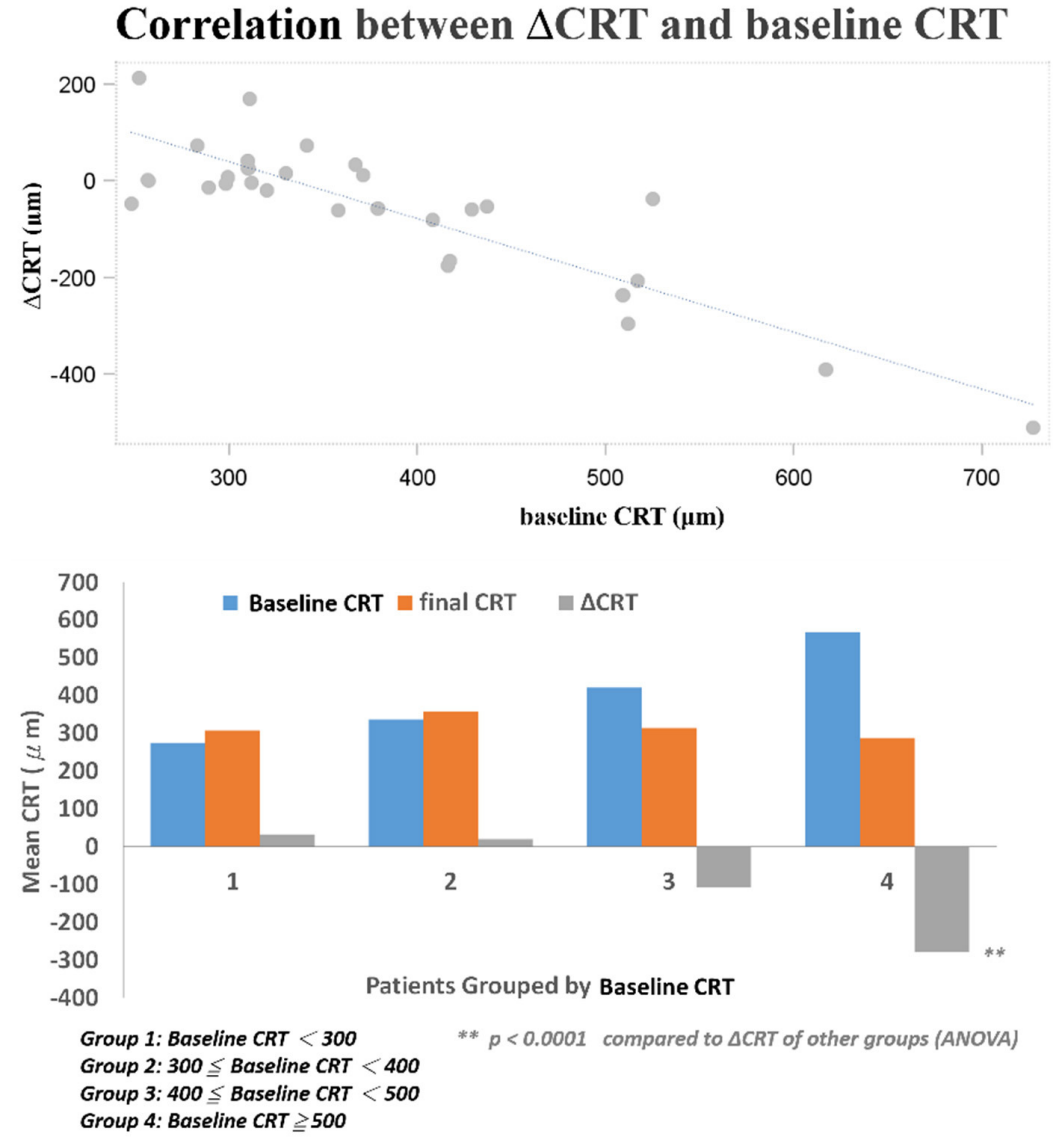
Correlation between changes in CRT and baseline CRT.

**Figure 4:**
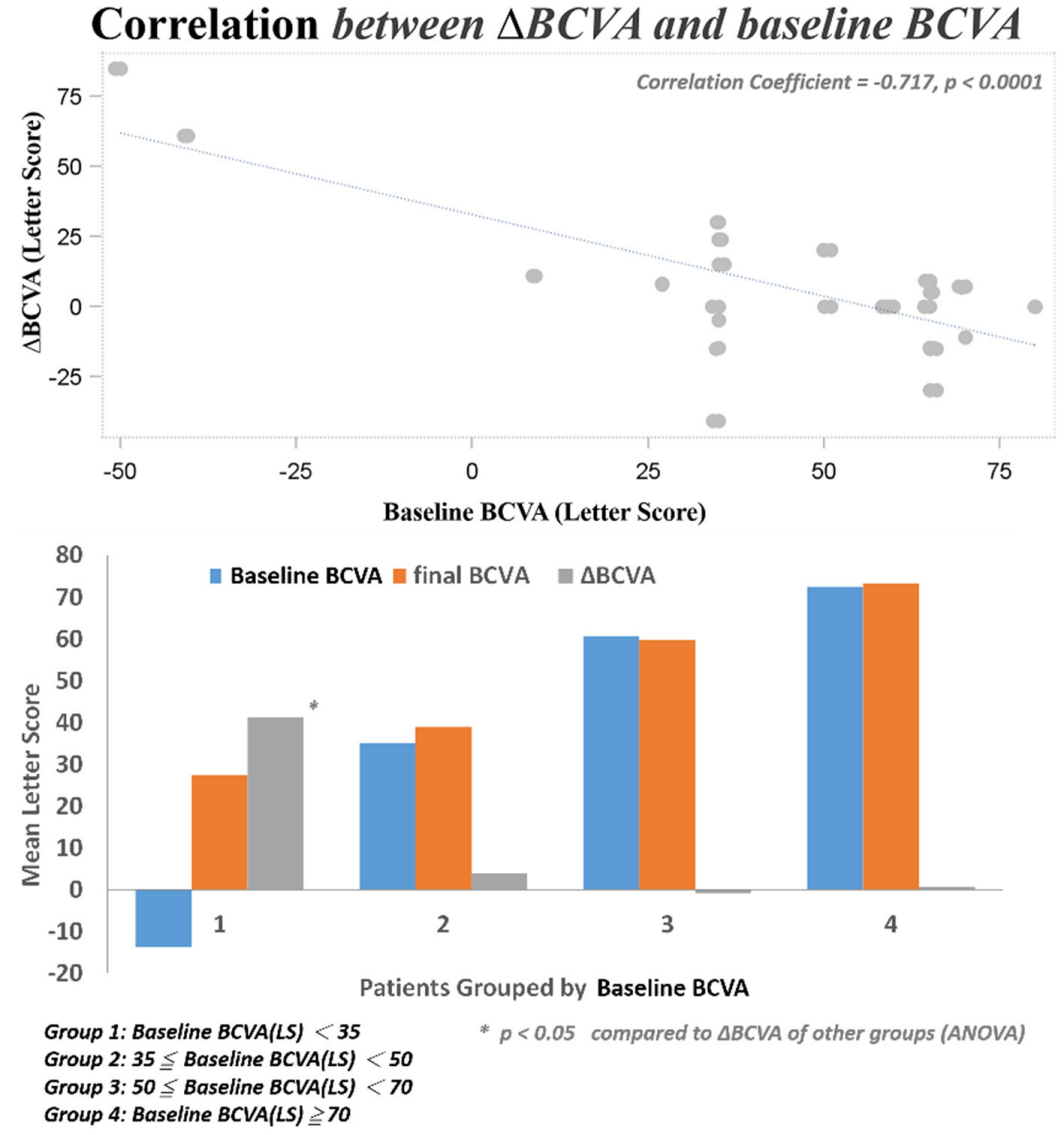
Correlation between changes in BCVA and baseline BCVA.

Several baseline patient parameters were analyzed to explore the correlation with the treatment responses (Table 2). Thicker baseline CRT (Figure 3), lower HbA1c, and worse BCVA (Figure 4) had better responses to the treatment. Multivariate logistic regression and general linear model analyses confirmed the same results that thicker baseline CRT and worse baseline BCVA had better responses to the treatment (*p* < 0.0001).

**Table 2:** Clinical parameters of patients with different therapeutic responses.

	**ΔCRT ≤ −50** ***n* = 12 (41.38%)**	**ΔCRT > −50** ***n* = 17 (58.62%)**	***p***	**ΔBCVA ≥ 15** ***n* = 8 (27.59%)**	**ΔBCVA< 15** ***n* = 21 (72.41%)**	***p***
Initial CRT 2	477.17 ± 106.90	319.00 ± 63.61	<0.0001^*^	420.38 ± 134.10	370.76 ± 106.37	0.305
Initial HbA1c	6.65 ± 0.59	7.99 ± 1.61	0.018^*^	7.12 ± 1.41	7.60 ± 1.50	0.487
Age	61.17 ± 10.25	61.94 ± 7.82	0.819	57.25 ± 6.09	63.29 ± 9.13	0.097
Gender						
Female	7 (58.33%)	6 (35.29%)	0.274	6 (75.00%)	7 (33.33%)	0.092
Male	5(41.67%)	11 (64.71%)		2 (25.00%)	14 (66.67%)	
Initial BCVA (Letters)	37.42 ± 27.27	50.24 ± 31.89	0.269	18.63 ± 40.19	54.95 ± 18.24	0.002^*^
Initial IOP	14.67 ± 2.93	15.12 ± 3.33	0.709	14.13 ± 3.00	15.24 ± 3.19	0.402
Anti-VEGF injection times	9.00 ± 4.43	7.53 ± 4.32	0.379	9.88 ± 3.80	7.48 ± 4.45	0.189
Lens status						
Phakic	9 (75.00%)	11 (64.71%)	0.694	7 (87.50%)	13 (61.90%)	
Pseudophakic	3 (25.00%)	6 (35.29%)		1 (12.50%)	8 (38.10%)	

* *p < 0.05; BCVA, best-corrected visual acuity; CRT, central retina thickness; HbA1c, glycated hemoglobin; IOP, intraocular pressure; VEGF, vascular endothelial growth factor*.

### Safety Outcomes

The mean final IOP (15.3 ± 3.2 mmHg) was not significantly higher than the baseline value (14.9 ± 3.1 mmHg, *p* = 0.5643), and not significantly higher than the IOP at 1 month after the last injection of ranibizumab (15.3 ± 3.3, *p* = 0.9985). The mean maximal IOP (the highest IOP recorded during follow-up) was 20.1 ± 4.7 mmHg, which was significantly higher than the baseline (14.9 ± 3.1 mmHg, *p* < 0.0001), but not significantly higher than the IOP at 1 month after the last ranibizumab injection (20.0 ± 4.5, mmHg, *p* = 0.8480). During the study, seven patients experienced IOP increases >22 mmHg after DEX implant, but all these patients had IOP returned to ≤ 22 mmHg after being managed with topical IOP-lowering medications.

All patients tolerated the treatment well, and none experienced serious ocular (e.g., endophthalmitis, non-infectious endophthalmitis, vitreous hemorrhage, retinal tear, retinal detachment, or sustained IOP elevations) or systemic adverse events during the follow-up period.

## Discussion

This retrospective case series, carried out in a tertiary medical center in central Taiwan, studied the therapeutic effects of DEX implant treatment on DME in eyes that had been unsuccessfully treated with intravitreal ranibizumab. In this study, DEX implants were effective in the anatomical improvement. Patients were assessed at monthly intervals postoperatively, and anatomic improvements as gauged by CRT were sustained throughout the entire course of follow-up. Even though improvements in CRT did not correlate with significantly improved BCVA, neither did BCVA decrease over the course of treatment and follow-up. DEX implants were well-tolerated, with only a few cases of increased IOP that were manageable with antihypertensive eyedrops.

Currently, there is no optimal treatment regimen for DEX implant therapy for DME (12). In the MEAD study, the protocol allows as-needed (*pro re nata*, PRN) retreatment with DEX implant with a frequency of no more than once every 6 months (13). As the out-of-pocket expense for our patients was about 40,000 NTD (1,370 USD) for each DEX implant during the study, we treated most eyes with one dose of DEX implant, followed by PRN injections when macular edema reoccurred. During the mean follow-up of 7.4 ± 4.6 months, almost 80% of the patients received only one DEX implant, and only two eyes received three injections.

The various available treatments for DME include anti-VEGFs, laser, surgery, and corticosteroids, with each targeting different pathogenic mechanisms of the disease (4). Our study suggests two main explanations for the observed benefit of DEX implant after refractory ranibizumab treatment: the pharmacologic and pharmacokinetic properties of the DEX implant and the possible tachyphylaxis or tolerance to ranibizumab. First, inflammation plays a prominent role in the pathogenesis of DME. Many features of inflammation, such as the leukocyte recruitment and adhesion to vascular endothelium (leukostasis), increased blood flow and vascular permeability, tissue (macular) edema, neovascularization, and upregulation of inflammatory mediators, have been described in both human and animal models of diabetic retinopathy (12, 14–18). Intravitreally administered corticosteroids act to ameliorate DME in multiple ways. As established anti-inflammatory agents, they reduce the production of pro-inflammatory factors, limit vascular permeability, and inhibit the expression of VEGFs (19). The DEX implant, a sustained-release drug delivery system for the potent corticosteroid dexamethasone, was developed to reduce the need for frequent intraocular injections due to the short half-life of intravitreally injected dexamethasone (<4 h) (20). The implant releases DEX into the vitreous for up to 6 months (21). In a previous study, Lazic et al. demonstrated the therapeutic efficacy of DEX implant for DME resistant to the anti-VEGF bevacizumab (22). The use of bevacizumab for DME is off-label, and therefore we examined patients who were initially treated with ranibizumab, which is FDA-approved for DME. Second, the patients' tachyphylaxis/tolerance to the previously administered ranibizumab might be another possible mechanism for the observed therapeutic effect after switching to intravitreal DEX implant. Even though there is a difference between tachyphylaxis and tolerance, both terms have long been presented as phenomena of reduced drug efficacy and are used synonymously in the literature (23). Tachyphylaxis/tolerance in chronic treatment with bevacizumab and ranibizumab was first described in 2007 for age-related macular degeneration (24, 25). Tachyphylaxis/tolerance to ranibizumab might be a result of the neutralization of ranibizumab by the formation of circulating antibodies, the desensitization of the target tissue to the drug, or the reactivation of DME driven by another pathway (26, 27). These effects might be circumvented with the use of pharmaceuticals aiming at other DME-associated pathways.

In our study, DEX implant treatment showed a generally favorable safety profile. Historically, adverse events most commonly associated with corticosteroid therapy include cataracts and steroid-induced glaucoma. Although none of our patients received a cataract surgery after DEX implants, the mean follow-up period was about 7 months, which may not be sufficient for the worsening of cataracts. Some patients in this study experienced transient increase in IOP that were successfully managed with topical medication. There was no case of serious ocular or systemic adverse events such as endophthalmitis, non-infectious endophthalmitis, vitreous hemorrhage, retinal tear, retinal detachment, or sustained IOP elevations.

We found three clinical factors that correlated with the treatment responses. Patients with lower baseline HbA1c had better anatomic improvement after treatment. The importance of glycemic control in the management of diabetic retinopathy was emphasized by previous studies (28, 29). The other two baseline predictive factors were thicker CRT and worse BCVA. Both factors correlated with better responses to DEX treatment in their respective aspects. Campos et al. also found that lower baseline BCVA predicted a higher visual acuity gain (30). It may be suggestive of a “ceiling effect” that cannot be ruled out completely in this prediction model; that is, smaller improvements are required to achieve good vision in patients with better starting vision, while those with lower BCVA at baseline have greater capacity to achieved better vision outcome. Another limit of visual acuity improvement was the uncertain optimal timing for DEX switching in patients with DME non-responder to intravitreal ranibizumab. If we can determine patients with DME who are more response to DEX implants, early switch to DEX implant may be performed, which may additional improve their final visual acuity. Limitations of this study include the small sample size and short-term follow-up, the uncontrolled retrospective design of the study, the non-standard treatment protocols, and a lack of consistent performance of fluorescein angiography prior to switching to DEX implant treatment. Nevertheless, this study showed that intravitreal DEX implant treatment was effective immediately after switch and safe in cases of refractory DME resistant to ranibizumab. Switching to DEX implant can be considered in eyes with DME that do not respond to anti-VEGF treatments. Furthermore, higher baseline CRT and worse BCVA were found to be the predictive factors for better therapeutic responses. However, further studies are necessary to determine the optimal timing for DEX switching in patients with DME non-responder to intravitreal ranibizumab and to shed light on the long-term outcomes of this treatment modality.

## Conclusion

This study demonstrated the feasibility of switching to intravitreal DEX implant in cases of DME that are refractory to intravitreal ranibizumab treatment. Conversion to DEX implant treatment resulted in a significant improvement in CRT. Although BCVA decreased a little after DEX treatment compared with BCVA after anti-VEGF injections, there was no statistical significance. Nevertheless, higher baseline CRT and worse BCVA can predict better therapeutic responses. Further larger-scale or multicenter studies would be conducted to explore different DEX treatment strategies for DME, such as first-line or early switch therapy, for better BCVA improvement.

## Data Availability Statement

The original contributions presented in the study are included in the article/supplementary material, further inquiries can be directed to the corresponding author/s.

## Ethics Statement

The studies involving human participants were reviewed and approved by China medical university and hospital research ethics committee-CMUH109-REC3-158. Written informed consent for participation was not required for this study in accordance with the national legislation and the institutional requirements. Written informed consent for publication of his clinical details and clinical images was obtained from the patient.

## Author Contributions

N-YH, C-JL, H-SC, C-HC, C-TL, J-ML, W-LC, P-TT, and Y-YT were responsible for substantial contributions to the conception or design of the work, and acquisition of data. C-JL, H-SC, and C-HC were responsible for interpretation of results. N-YH, C-JL, and HB participated in the design and was a major contributor in writing the manuscript. N-YH, C-JL, HB, W-LC, and W-CW were responsible for final approval of the version to be published. All authors reviewed and approved the final manuscript.

## Conflict of Interest

H-SC is employed by company NephroCare Ltd. The remaining authors declare that the research was conducted in the absence of any commercial or financial relationships that could be construed as a potential conflict of interest.

## Abbreviations

DEX: dexamethasone
DME: diabetic macular edema
BCVA: best-corrected visual acuity
CRT: central retinal thickness
IOP: intraocular pressure
VEGF: vascular endothelial growth factor
BRB: blood-retinal barrier
FDA: Food and Drug Administration
HbA1c: glycated hemoglobin
ETDRS: Early Treatment Diabetic Retinopathy Study
GLMM: generalized linear mixed model.
